# Identification of Therapeutic Targets for Amyotrophic Lateral Sclerosis Using PandaOmics – An AI-Enabled Biological Target Discovery Platform

**DOI:** 10.3389/fnagi.2022.914017

**Published:** 2022-06-28

**Authors:** Frank W. Pun, Bonnie Hei Man Liu, Xi Long, Hoi Wing Leung, Geoffrey Ho Duen Leung, Quinlan T. Mewborne, Junli Gao, Anastasia Shneyderman, Ivan V. Ozerov, Ju Wang, Feng Ren, Alexander Aliper, Evelyne Bischof, Evgeny Izumchenko, Xiaoming Guan, Ke Zhang, Bai Lu, Jeffrey D. Rothstein, Merit E. Cudkowicz, Alex Zhavoronkov

**Affiliations:** ^1^Insilico Medicine Hong Kong Ltd., Hong Kong Science and Technology Park, Hong Kong, Hong Kong SAR, China; ^2^Department of Neuroscience, Mayo Clinic Florida, Jacksonville, FL, United States; ^3^College of Clinical Medicine, Shanghai University of Medicine and Health Sciences, Shanghai, China; ^4^International Center for Multimorbidity and Complexity in Medicine (ICMC), Universität Zürich, Zurich, Switzerland; ^5^Department of Medicine, Section of Hematology and Oncology, University of Chicago, Chicago, IL, United States; ^6^4B Technologies Limited, Suzhou BioBay, Suzhou, China; ^7^Neuroscience Graduate Program, Mayo Clinic Graduate School of Biomedical Sciences, Jacksonville, FL, United States; ^8^School of Pharmaceutical Sciences, IDG/McGovern Institute for Brain Research, Tsinghua University, Beijing, China; ^9^Brain Science Institute, Johns Hopkins University School of Medicine, Baltimore, MD, United States; ^10^Department of Neurology, Johns Hopkins University School of Medicine, Baltimore, MD, United States; ^11^Healey & AMG Center for ALS, Massachusetts General Hospital, Harvard Medical School, Boston, MA, United States; ^12^Buck Institute for Research on Aging, Novato, CA, United States

**Keywords:** target discovery, target novelty, artificial intelligence, time machine, multi-omics

## Abstract

Amyotrophic lateral sclerosis (ALS) is a severe neurodegenerative disease with ill-defined pathogenesis, calling for urgent developments of new therapeutic regimens. Herein, we applied PandaOmics, an AI-driven target discovery platform, to analyze the expression profiles of central nervous system (CNS) samples (237 cases; 91 controls) from public datasets, and direct iPSC-derived motor neurons (diMNs) (135 cases; 31 controls) from Answer ALS. Seventeen high-confidence and eleven novel therapeutic targets were identified and will be released onto ALS.AI (http://als.ai/). Among the proposed targets screened in the c9ALS *Drosophila* model, we verified 8 unreported genes (*KCNB2*, *KCNS3*, *ADRA2B*, *NR3C1*, *P2RY14*, *PPP3CB*, *PTPRC*, and *RARA*) whose suppression strongly rescues eye neurodegeneration. Dysregulated pathways identified from CNS and diMN data characterize different stages of disease development. Altogether, our study provides new insights into ALS pathophysiology and demonstrates how AI speeds up the target discovery process, and opens up new opportunities for therapeutic interventions.

## Introduction

Amyotrophic lateral sclerosis (ALS) is a rare neuromuscular disease resulting from progressive degeneration of motor neurons (MNs) in the brain and spinal cord. With involvement of celebrities, politicians, and athletes worldwide, the Ice Bucket Challenge in 2014 successfully promoted the awareness of this devastating illness. It is the most common MN disease (Roman, 1996), with the incidence ranging from 0.6 to 3.8 per 100,000 person-years (Longinetti and Fang, 2019). Approximately 16,500 ALS cases were diagnosed in the United States in 2015 (Mehta et al., 2018a). The onset of the disease typically occurs in middle adulthood, with a mean survival time hovering at 3-5 years post diagnosis (Chio et al., 2009). Although the signs and symptoms of ALS vary due to the difference in the region of neurons being affected, patients usually experience painless progressive muscle weakness and paralysis. ALS is an age-related disease, with the prevalence expected to increase with population aging (Mehta et al., 2018b). It is reported that aging induces damaged protein accumulation, oxidative stress, disrupted energy homeostasis, and DNA damage, reducing the viability of the affected neurons (Hou et al., 2019).

ALS can be categorized based on its root causes - familial or sporadic (Grad et al., 2017). Familial ALS (fALS) contributes to 10% of the cases and involves mutations in specific genetic loci that are inherited in an autosomal dominant manner (Alsultan et al., 2016). Over 20 genetic risk factors are identified for fALS (Souza et al., 2015). Notably, *SOD1*, *TARDBP*, *C9orf72*, and *FUS* have been extensively characterized. According to a pooled summary of mutation frequency in 111 studies, those four major ALS-associated genes explain 47.7% fALS and 5.2% sporadic ALS (sALS) cases (Zou et al., 2017), leaving a substantial fraction of the genetic basis of ALS undiscovered. Given the heterogeneous genetic involvement in ALS, several pathophysiological mechanisms have been proposed, including aberrant proteostasis, altered RNA metabolism, nucleocytoplasmic transport defects, mitochondrial dysfunction, DNA repair deficiency, axonal transport defects, vesicle transport dysregulation, excitotoxicity, oligodendrocyte dysfunction, and neuroinflammation (Mejzini et al., 2019).

Presently, ALS remains an incurable disease due to an inadequate understanding of disease mechanisms. The United States Food and Drug Administration (FDA) has approved four drugs for the treatment of ALS, including Riluzole, Tiglutik, Edaravone, and Nuedexta. Riluzole - an inhibitor of sodium channel α subunit - is the first FDA-approved neuroprotective agent for ALS and the only drug that prolongs the survival of ALS patients. Beside blocking the glutamatergic transmission, riluzole has a wide range of neural effects, including inhibition of persistent and fast sodium currents, suppression of neurotransmitter release, diminishment of voltage-gated calcium and potassium currents, and potentiation of calcium-dependent potassium current (Doble, 1996; Bellingham, 2011). These are possible explanations for the modest efficacy of riluzole in extending patients’ survival (Riviere et al., 1998). Tiglutik, the oral suspension formulation of riluzole, was designed for ALS patients with difficulties in swallowing. Edaravone is a free radical scavenger against reactive oxygen species-driven MN death and inflammation (Jami et al., 2015; Watanabe et al., 2018). It was approved for marketing and manufacturing in Japan in 2015, and received FDA-approval as an ALS treatment in 2017. Clinically, edaravone demonstrates its potent antioxidant property by reducing peroxynitrite and its association with neurotoxin in cerebrospinal fluid (Yoshino and Kimura, 2006) and plasma (Nagase et al., 2016) of ALS patients, respectively. Although significant improvement in ALS Functional Rating Scale-Revised (ALSFRS-R) score is reported in edaravone-treated ALS patients (Writing Group and Edaravone (MCI-186) ALS 19 Study Group, 2017; Takahashi et al., 2017), its long-term efficacy remains questionable and requires additional trials for confirmation. Nuedexta is an oral medication containing dextromethorphan and quinidine. Phase 3 studies have demonstrated its efficacy in reducing the frequency and severity of pseudobulbar affect in ALS patients (Brooks et al., 2004; Pioro et al., 2010).

**FIGURE 1:**
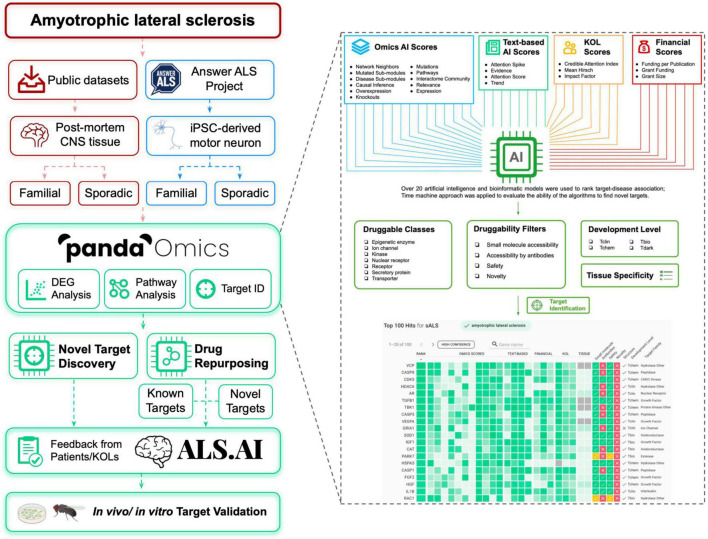
Flowchart for ALS target discovery and drug repurposing. Target identification was performed with the public CNS tissue-based datasets, and diMN data from Answer ALS on PandaOmics. Targets were divided into two categories: novel targets for further investigation and targets for drug repurposing. The targets will be released onto ALS.AI. Feedback on proposed targets will be collected from ALS KOLs to select the best candidates for further validation. The identified targets will be further validated using *in vivo* and *in vitro* models. The combined usage of PandaOmics and ALS.AI significantly reduces the time required for novel target discovery and drug investigation for ALS treatment, which points to a potential direction to search for the treatment of other human diseases.

Thanks to the advancements in genomic profiling techniques, numerous genome-wide association studies have screened for common genetic variants in ALS and have identified novel candidates as either genetic risk factors or biomarkers (e.g., *ACSL5*, *KIF5A*, *ATXN2*, and *MOBP*) (van Rheenen et al., 2016; Gibson et al., 2017; Nicolas et al., 2018; Nakamura et al., 2020). Genomic profiling of central nervous system (CNS) tissues and blood from ALS patients may also assist in uncovering the differentially expressed genes that contribute to disease-driving mechanisms (Dangond et al., 2004; Zhang et al., 2011; Riva et al., 2016; Swindell et al., 2019). Furthermore, the utility of both cellular and animal models with ALS-linked gene variants helps to determine the potential interacting partners of those ALS-linked genes, providing multiple lines of evidence for uncovering disease pathology (Jeong et al., 2011; Milanese et al., 2014; Fujisawa et al., 2016). Here, we applied PandaOmics, an artificial intelligence (AI)-powered target discovery platform, to explore dysregulated expression of genes and altered pathways across various ALS-related datasets with a goal to identify potential therapeutic targets. As illustrated in Figure 1, we utilized post-mortem CNS tissues and direct iPSC (induced pluripotent stem cell)-differentiated motor neurons (diMN) derived from ALS patients to perform target discovery. Using over 20 AI and bioinformatics models, PandaOmics ranks targets based on their target-disease associations as well as information on druggability, developmental state and tissue specificity. By customizing different filter settings, 17 high-confidence and 11 novel candidates (28 in total) were selected as potential ALS therapeutic targets. Proposed targets will be released onto the platform ALS.AI^1^. To evaluate the utility of this approach, proposed candidates were validated in a *Drosophila* model mimicking *C9orf72*-mediated ALS (c9ALS), the most common fALS case. The aim of this study is to demonstrate the utilization of the AI-driven target discovery platform – PandaOmics – to identify therapeutic targets for ALS.

## Materials and Methods

### Data Sources and Availability

Microarray and RNA sequencing (RNA-seq) datasets for ALS patients and control samples were retrieved from public repositories and processed by PandaOmics for downstream analysis and target identification. Over 60 ALS-related datasets of various tissue sources were available in PandaOmics (Supplementary Figure 1), including datasets of post-mortem CNS tissues, iPSC-derived neurons, blood, etc. For each dataset, samples could be divided into subgroups based on their clinical subtypes or other phenotypic attributes. In addition, transcriptomic and proteomic data of the diMNs, generated from ALS patients and neurologically healthy subjects in Answer ALS (Baxi et al., 2022) were uploaded to PandaOmics and incorporated in our analyses.

The raw transcriptomic data of CNS comparisons were available in public repositories, which could be retrieved by their series identifiers. In addition, transcriptomic and proteomic profiles of the diMN samples were available to investigators upon request and approval from Answer ALS.

### Answer ALS

At present, Answer ALS is the largest collaborative effort in ALS bringing together multiple research organizations and key opinion leaders (KOLs). Over eight hundred ALS patients and one hundred healthy controls from eight neuromuscular clinics distributed across the United States were enrolled in this project. Blood sample was collected at the first visit of each participant. The iPSC lines were generated from peripheral blood mononuclear cells extracted from whole blood *via* an episomal iPSC reprogramming system. The iPSCs underwent three major stages of differentiation for 32 days to generate the mixture of mature motor neuron populations. Detailed protocol for the diMN generation and standards of quality assurance were described by Baxi et al. (2022). The consortium generated multi-omics data comprising genomic, epigenomic, transcriptomic, proteomic, laboratory test, medical records and other data. A data portal was established and open to both academia and industry.

### Dataset and Comparison Selection

Given that the degeneration of motor neurons in the brain and spinal cord underlay ALS pathogenesis, CNS tissue datasets were selected for analysis in the present study. Since the family history of ALS occurrence was not available for the CNS datasets, patients were classified into different subtypes based on their genotypes. Samples carrying one of the four major fALS-linked gene variations (*SOD1*, *TARDBP*, *FUS*, and *C9orf72*) were classified as the fALS group, and those with other or unspecified gene variations as the sALS group, yielding five independent fALS as well as seven independent sALS case-control comparisons (Table 1).

**TABLE 1:** ALS case-control comparisons using CNS and diMN samples.

Subtype	Data series	Platform	Technology	Source	Mutant gene	# Case	# Control	Year	References
***CNS comparisons***									
fALS	E-MTAB-1925	A-MEXP-2246	Microarray	Motor Cortex	*C9orf72*	3	3	2013	Donnelly et al., 2013
fALS	GSE67196	GPL11154	RNA-seq	Cerebellum	*C9orf72*	8	8	2015	Prudencio et al., 2015
fALS	GSE67196	GPL11154	RNA-seq	Frontal Cortex	*C9orf72*	8	9	2015	Prudencio et al., 2015
fALS	GSE68605	GPL570	Microarray	Motor Neurons	*C9orf72*	8	3	2015	Cooper-Knock et al., 2015
fALS	GSE20589	GPL570	Microarray	Motor Neurons	*SOD1*	3	7	2010	Kirby et al., 2011
sALS	GSE122649	GPL18573	RNA-seq	Motor Cortex	–	26	12	2018	Tam et al., 2019
sALS	GSE124439	GPL16791	RNA-seq	Frontal Cortex	–	65	9	2018	Tam et al., 2019
sALS	GSE124439	GPL16791	RNA-seq	Motor Cortex	–	80	8	2018	Tam et al., 2019
sALS	GSE19332	GPL570	Microarray	Motor Neurons	–	3	7	2009	Cox et al., 2010
sALS	GSE76220	GPL9115	RNA-seq	Spinal Motor Neurons	–	13	8	2015	Krach et al., 2018
sALS	GSE67196	GPL11154	RNA-seq	Cerebellum	–	10	8	2015	Prudencio et al., 2015
sALS	GSE67196	GPL11154	RNA-seq	Frontal Cortex	–	10	9	2015	Prudencio et al., 2015
***diMN transcriptomic comparisons***									
fALS	Answer ALS	Novaseq 6000	RNA-seq	diMN	–	25	31	2022	Baxi et al., 2022
sALS	Answer ALS	Novaseq 6000	RNA-seq	diMN	–	110	31	2022	Baxi et al., 2022
***diMN proteomic comparisons***									
fALS	Answer ALS	SCIEX 6600	SWATH-MS	diMN	–	25	31	2022	Baxi et al., 2022
sALS	Answer ALS	SCIEX 6600	SWATH-MS	diMN	–	110	31	2022	Baxi et al., 2022

The non-Hispanic and non-Latino whites represented the largest ethnic group in the datasets from Answer ALS, amounting to over 85% of the total samples. In this regard, diMN samples belonging to this ethnic group with both transcriptomic and proteomic data were selected for the current analysis. The samples were further divided into 25 fALS and 110 sALS based on the presence or absence of the family history of ALS occurrence. As a result, two subtype-dependent comparisons were built using the diMN transcriptomic and proteomic data, respectively (Table 1).

### Meta-Analysis

To identify potential targets for ALS, all case-control comparisons belonging to the same comparison groups (two CNS groups: five fALS and seven sALS transcriptomic comparisons; four diMN groups: fALS transcriptomic, sALS transcriptomic, fALS proteomic, and sALS proteomic comparisons) were pooled into a single meta-analysis, yielding a total of six meta-analyses. An illustration of the target identification process is available in Supplementary Figure 2.

### PandaOmics Scores

The advance of generative adversarial networks (GANs) accelerated the process of target discovery using transcriptomic data and *de novo* molecular design (Aliper et al., 2016; West et al., 2018; Vanhaelen et al., 2020). PandaOmics was a cloud-based target discovery platform that incorporated multiple scores developed using transcriptomic and proteomic data, text data including grants, scientific literature, publications, patents, stock reports, molecular data, as well as multiple meta-data repositories. It was accessible on a software-as-a-service (SaaS) basis at https://pandaomics.com. PandaOmics utilized advanced deep learning models and AI approaches to predict the target genes associated with a given disease through a combination of Omics AI scores, Text-based AI scores, Finance scores, and KOL scores (Supplementary Table 1). In addition, Druggability filters, Tissue specificity filters, Target family filters, and Development filters could be applied to further refine the list to meet the user’s research goals. The AI and bioinformatic models were validated with our “time machine” approach, enabling unique and flexible therapeutic target hunting workflows. During the target identification process, our AI models dynamically assessed disease targets based on a variety of measures, such as novelty, accessibility by small molecules and biologics, safety and tissue expression specificity, to collectively generate hypotheses around their potential druggability profiles. Previous studies demonstrated the effectiveness of PandaOmics to identify novel and repurposing therapeutic targets (Insilico Medicine, 2022; Pun et al., 2022; Vera et al., 2022).

### Validation of the Scoring Approach

The “time machine” approach was applied for the validation of the ability of a model to identify the truly novel targets of the disease of interest. The data before a given year was used as training data and the trained model was then evaluated based on the targets entering the clinical trial after the given year (Supplementary Figure 3A). Two validation metrics were used to validate the scoring approach. Log fold change of enrichment **(ELFC)** referred to the log-transformed fold change of enrichment showing how much the top of the list was enriched by known targets, and was calculated by formula (I):


(I) $$E\,L\,F\,C\,\left(s\,c\,o\,r\,e\right)=l\,o\,g_{2}\,\left(\frac{t\,a\,r\,g\,e\,t\,s_{k}\cdot N}{k\cdot t\,a\,r\,g\,e\,t\,s_{N}}\right)$$

where targets_*k*_ was the number of known targets for this disease in top-k (or 0.1 if there were none), and targets_*N*_ was the total number of known targets for this disease among the genes that were available for a particular PandaOmics score. And hypergeometric *p*-value **(HGPV)** stood for the statistical significance of the effect and showed how likely the same level of enrichment could be achieved from the random distribution and was calculated by formula (II):


(II) $$H\,G\,P\,V\,\left(s\,c\,o\,r\,e\right)=-l\,o\,g_{10}\,\left(1-h\,g\,c\,d\,f\,\left(t\,a\,r\,g\,e\,t\,s_{k},k,t\,a\,r\,g\,e\,t\,s_{N},N\right)\right)$$

where hgcdf was a hypergeometric cumulative distribution function. A score with higher values of **ELFC** and **HGPV** corresponded to the higher predictive power of the target-disease association (Supplementary Figure 3B).

### Filter Setting for Target Identification

To find high-confidence targets, all Omics and Text-based scores were employed, along with the Grant Funding score which was the most representative in the Financial category, and two KOL scores (credible attention index and impact factor) that prioritized the targets based on literature evidence in high-quality journals. To make sure the identified targets were actionable, the Druggable Class filter was switched on. Simultaneously, we customized the Druggability filters to screen targets already associated with small molecules and not considered as essential genes in the Online GEne Essentiality database (Supplementary Figure 4). A list of high-confidence druggable targets was ranked in descending order based on their metascores, and the top-50 targets were selected for further investigation.

Similarly, novel ALS targets were identified without prior knowledge by restricting the Druggability filter to a higher novelty level, selecting only the Omics scores, and disabling the Text-based, Financial, and KOL scores (Supplementary Figure 5). After recalculating the metascores with the new criteria, the top-50 ranked genes were selected as novel targets for further analysis.

### Pathway Analysis

The schematic representation of pathway analyses conducted in the present study is shown in Supplementary Figure 6. The degree of pathway dysregulation was determined by the PandaOmics proprietary iPANDA algorithm accounting for the differential gene expression and the topological decomposition of pathways (Ozerov et al., 2016), which was adopted in pathway activation scoring (Makarev et al., 2014; Zhu et al., 2015). We analyzed all the CNS and diMN comparison groups for pathway dysregulation based on Version 73 of the Reactome database (Jassal et al., 2020). For each group, a pathway was considered as dysregulated when 1) its alteration was unidirectional in greater than or equal to 80% of all the comparisons of the ALS subtype, and 2) the absolute iPANDA value reached the threshold of 0.01 in at least one comparison of a subtype. Networks of dysregulated pathways were constructed using EnrichmentMap (Merico et al., 2010) in Cytoscape (Shannon et al., 2003). The hierarchical level of pathways retrieved from the Reactome database was employed as the basis for the annotation of pathway clusters in the networks. The dysregulated pathways of each group were further evaluated for their enrichment in each of the top-level biological processes of Reactome hierarchy using hypergeometric tests by formula (III):


(III) $$p=1-\sum_{i=0}^{r-1}\frac{\left(\frac{K}{i}\right)\,\left(\frac{N-K}{n-i}\right)}{\left(\frac{N}{n}\right)}$$

where ***N*** stood for the total number of pathways defined in Reactome database, ***K*** represented the number of dysregulated pathways in the interested biological process, ***n*** was the total number of dysregulated pathways in a comparison group, and ***r*** represented the number of pathways belonging to the interested biological process. The *p*-values were adjusted by Bonferroni correction for multiple testing.

### *Drosophila* Genetics and Eye Degeneration Scoring

All flies were raised at 25°C on the regular yeast-cornmeal-molasses diet. Flies expressing expanded G_4_C_2_ repeats were generated by injecting pUAST plasmids with 30 G_4_C_2_ repeats in a *w*^1118^ strain. Details were discussed in Xu et al. (2013). For genetic screens, *GMR-GAL4, UAS-(G_4_C_2_)_30_/CyO, twi-GAL4, UAS-GFP* was crossed to *UAS-RNAi* against genes of interest. Non-CyO offspring were collected and aged for 15 days. Eye degeneration was scored using a method described in Zhang et al. (2015) based on the disruption in the external morphology of the eye with positive or negative scores corresponding to an increase or decrease in severity. A modification score ranging from −4 to 2 was used to describe the relative severity of the morphology defect based on the following phenotypes: amount and orientation of supernumerary interommatidial bristles, necrotic patches, retinal collapse, size, ommatidial structure, and degree of depigmentation.

## Results

### Potential Therapeutic Targets

In the present study, 12 CNS-based comparisons and 4 diMN-based comparisons (Table 1) of ALS patients and healthy controls were subjected to 6 corresponding meta-analyses, generating 12 target lists with two levels of novelty for detailed target evaluation. Potential therapeutic targets for ALS were selected based on their ranking calculated by PandaOmics, consistency of the dysregulated expression across different comparisons, druggability, safety assessment, and clinical trial status, yielding a list of 28 potential candidates (Table 2). Seventeen high-confidence and eleven novel therapeutic targets were identified from post-mortem CNS tissue- and diMN-derived data. All selected targets belonged to the druggable classes defined by PandaOmics, with supportive evidence on either ALS or neurodegeneration, and ranked as the top-50 targets in at least one of the meta-analyses. For CNS targets, they were consistently upregulated or downregulated in at least 80% of all comparisons for fALS, sALS or both. Selected promising targets whose suppression led to the most notable rescue of degenerations in the c9ALS *Drosophila* model are discussed below.

**TABLE 2:** List of potential therapeutic targets.

Gene^1^	fALS^2^	sALS^2^	Protein family	Tissue enrichment^3^	Proposed ALS mechanism
***High-confidence (CNS)***					
*ADRA2B*	80%	50%	GPCR	Low tissue specificity	Protein degradation
*CYBB*	80%	86%	Ion channel	Blood, lung, lymphoid tissue	Oxidative stress
FLT1*	80%	57%	Receptor kinase	Placenta	Inflammation
MAP3K5*	80%	71%	Protein kinase	Adrenal gland	Apoptosis
*MAPK1*	80%	71%	CMGC kinase	Brain	Apoptosis
*NOS1*	80%	86%	Oxidoreductase	Brain and skeletal muscle	Oxidative stress
NR3C1*	80%	86%	Nuclear receptor	Low tissue specificity	Inflammation, excitotoxicity
PTK2*	40%	86%	Tyrosine kinase	Low tissue specificity	Protein aggregation
*PTPRC*	80%	86%	Receptor phosphatase	Blood, lymphoid tissue	Inflammation
*RARA*	50%	14%	Nuclear receptor	Low tissue specificity	Neurogenesis
***Novel (CNS)***					
*AHCYL1*	100%	71%	Enzyme	Low tissue specificity	Apoptosis
*KCNB2*	60%	83%	Ion channel	Brain, lymphoid tissue, pituitary gland	Excitotoxicity
*P2RY14*	40%	14%	GPCR	Granulocytes, dendritic cells, placenta	Inflammation
*SCYL1*	40%	14%	Protein kinase	Low tissue specificity	Apoptosis
*SLC25A10*	20%	29%	Transporter	Liver	Oxidative stress
STUB1*	20%	14%	Acyltransferase	Low tissue specificity	Protein degradation
***High-confidence (diMN)***					
*DNMT3A*	0.1324 (0.0346)	0.0773 (0.1172)	Methyltransferase	Low tissue specificity	Apoptosis
*ERN1*	0.2058 (0.003)	0.0699 (0.1644)	Protein kinase	Low tissue specificity	Protein aggregation, apoptosis
*G6PD#*	−0.1416 (0.0487)	−0.0847 (0.1337)	Oxidoreductase	Testis	Oxidative stress
HSPD1*#	0.1363 (0.1365)	0.1916 (0.0083)	Isomerase	Vagina	FUS pathology, inflammation
PPIA*#	0.1728 (0.0338)	0.2361 (0.0003)	Isomerase	Low tissue specificity	TDP-43 pathology, inflammation
*RPS6KB1*	0.1558 (0.0297)	0.1426 (0.0052)	AGC kinase	Low tissue specificity	Protein aggregation
VCP*#	0.0212 (0.637)	0.0776 (0.0411)	Hydrolase	Low tissue specificity	Mitochondrial dysfunction
***Novel (diMN)***					
*KCNS3*	0.3886 (0.0995)	0.3338 (0.0282)	Ion channel	Skeletal muscle	Excitotoxicity
*PPP3CB#*	−0.3048 (0.0115)	−0.1371 (0.1725)	Esterase	Skeletal muscle	Protein aggregate degradation
*PSMC6#*	−0.2636 (0.0402)	−0.1502 (0.0926)	Hydrolase	Low tissue specificity	Proteostasis
*METTL21A*	0.196 (0.0012)	0.0827 (0.0432)	Methyltransferase	Low tissue specificity	Protein aggregation
*TOPORS*	0.2161 (0.0135)	0.1385 (0.0184)	Acyltransferase	Low tissue specificity	Apoptosis

^1^Manually curated aging-associated genes (marked with *) based on clinical trials (https://clinicalTrials.gov/), publication, geroprotectors (http://geroprotectors.org) and GenAge database (https://genomics.senescence.info/genes/index.html); for the diMN targets, targets identified using proteomic data are marked with #;

*^2^Shown for CNS targets are percentages of comparisons with up-regulated target (LFC > 0) out of five fALS or seven sALS comparisons; Shown for diMN targets are LFC and p-value in parenthesis;*

*^3^Tissue enrichment (RNA) retrieved from Human Protein Atlas (https://www.proteinatlas.org/) on November 15, 2021.*

#### ADRA2B

**FIGURE 2:**
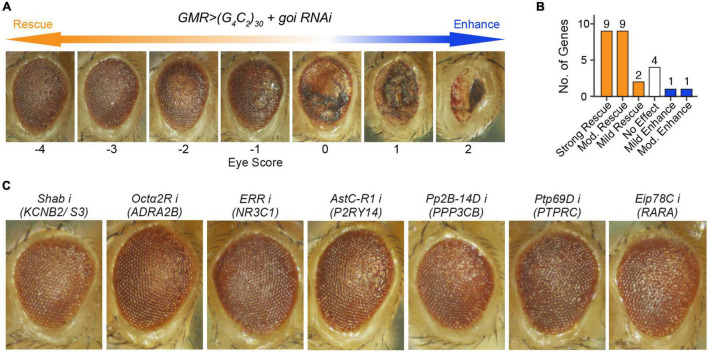
Loss of seven unreported fly orthologs, corresponding to eight genes, strongly rescued (G_4_C_2_)_30_-mediated neurodegeneration in a c9ALS *Drosophila* model. **(A)** A scale of magnitude of degeneration in fly eyes expressing (G_4_C_2_)_30_ scored from –4 to 2. The control flies (score 0), whose eyes expressing (G_4_C_2_)_30_, exhibited eye degeneration, as indicated by necrotic patches, loss of ommatidia, depigmentation, and retinal collapse. The degree of eye degeneration rescue by RNAi of the gene of interest (goi) ranged from –4 to 2, where score –4 represented the strongest degree of rescue and score 2 stood for the highest degree of enhancement. **(B)** Number of genes whose loss gave rise to different degrees of modifications. Strong rescue stood for a score ≤ –3, moderate (mod.) rescue or enhance for a score ≤ –2 or ≥ 2, mild rescue or enhance for a score ≤ –1 or ≥ 1, and no effect for a score > –1 and < 1. For a gene with multiple fly orthologs, the score corresponding to the strongest modification of eye degeneration was used to represent the effect of suppressing the gene. **(C)** Fly eyes expressing (G_4_C_2_)_30_ with RNAi against (*from left to right*) *Shab* (fly ortholog of *KCNB2* and *KCNS3*), *Octα2R* (fly ortholog of *ADRA2B*), *ERR* (fly ortholog of *NR3C1*), *AstC-R1* (fly ortholog of *P2RY14*), *Pp2B-14D* (fly ortholog of *PPP3CB*), *Ptp69D* (fly ortholog of *PTPRC*), and *Eip78C* (fly ortholog of *RARA*).

*ADRA2B* was upregulated in 80% of CNS transcriptomic fALS comparisons. Our c9ALS fly model showed that RNAi against *Octα2R* (fly ortholog of *ADRA2B*) ameliorated eye degeneration (Score = −3, Figure 2C and Table 3) indicating that suppressing the expression of *ADRA2B* may offer beneficial effects to fALS patients. Our findings were in line with the study reporting that treatment with ADRA2B agonist (rilmenidine) worsened motor neuron degeneration in SOD1^G93A^ mice (Perera et al., 2018). ADRA2B was connected with several available drugs. Both agonists and antagonists targeting ADRA2B were tested for multiple neurological diseases, such as bipolar disease, brain injury, and Parkinson’s disease (PD), but not ALS. As such, our findings suggested *ADRA2B* as a potential drug repurposing target for ALS.

**TABLE 3:** Screen results using a c9ALS *Drosophila* model.

Human symbol	Fly symbol	Availability of fly model	Score	Interpretation	References
*KCNB2*	*Shab*	Yes	−3.5	Strong rescue	–
*KCNS3*	*Shab*	Yes	−3.5	Strong rescue	–
*VCP*	*TER94*	Yes	−3.5	Strong rescue	Zhang et al., 2015
*ADRA2B*	*Octα2R*	Yes	−3	Strong rescue	–
*NR3C1*	*ERR*	Yes	−3	Strong rescue	–
*P2RY14*	*AstC-R1*	Yes	−3	Strong rescue	–
	*AstC-R2*	Yes	−2	Moderate rescue	–
*PPP3CB*	*Pp2B-14D*	Yes	−3	Strong rescue	–
	*CanA-14F*	Yes	−2	Moderate rescue	–
*PTPRC*	*Ptp69D*	Yes	−3	Strong rescue	–
*RARA*	*Eip78C*	Yes	−3	Strong rescue	–
	*EcR*	Yes	−2.5	Moderate rescue	–
	*Hr96*	Yes	−2	Moderate rescue	–
	*Eip75B*	Yes	Lethal	–	–
*PTK2*	*Fak*	Yes	−2.5	Moderate rescue	–
*AHCYL1*	*AhcyL1*	Yes	−2	Moderate rescue	–
*DNMT3A*	*ADD1*	Yes	−2	Moderate rescue	–
	*sba*	Yes	0	No modification	–
*ERN1*	*Ire1*	Yes	−2	Moderate rescue	–
*MAP3K5*	*Ask1*	Yes	−2	Moderate rescue	–
*MAPK1*	*rl*	Yes	−2	Moderate rescue	–
*SCYL1*	*yata*	Yes	−2	Moderate rescue	–
*STUB1*	*STUB1*	Yes	−2	Moderate rescue	–
*TOPORS*	*Topors*	Yes	−2	Moderate rescue	–
*G6PD*	*Zw*	Yes	−1	Mild rescue	–
*NOS1*	*Nos*	Yes	−1	Mild rescue	–
*HSPD1*	*Hsp60A*	Yes	−0.5	No modification	–
*CYBB*	*Duox*	Yes	0	No modification	–
*PSMC6*	*Rpt4*	Yes	0	No modification	Zhang et al., 2015
*PPIA*	*CG7768*	Yes	0.5	No modification	Neuro et al., 2021
	*Cyp1*	Yes	0.5	No modification	Neuro et al., 2021
*FLT1*	*Pvr*	Yes	1	Mild enhancement	Neuro et al., 2021
*RPS6KB1*	*S6k*	Yes	2	Moderate enhancement	–
*METTL21A*	*CG5013*	No	–	–	–
*SLC25A10*	*Dic1*	No	–	–	–

*Both KCNB2 and KCNS3 correspond to Shab in Drosophila. A score of −3.5 stood for the situation that some offspring flies were scored −4 and some were −3. The same applies to the scores of −2.5, −0.5 and 0.5.*

#### NR3C1

*NR3C1* was found to be generally upregulated in both CNS fALS (80%) and sALS (86%) comparisons. *NR3C1* encodes a glucocorticoid receptor with a dual role as a modulator of transcription factors and a transcription factor itself. Over 60 drugs are associated with *NR3C1*, including both agonists and antagonists. NR3C1 agonist and its ligand, glucocorticoids, were widely adopted as the standard treatment for inflammatory diseases (Escoter-Torres et al., 2019). However, none of the ALS patients reached the pre-defined responder criteria with the immunosuppression therapy involving two NR3C1 agonists, viz. methylprednisolone and prednisone (Fournier et al., 2018). In contrast, an NR3C1 antagonist (CORT113176) reduced the expression and origin of pro-inflammatory factors (Meyer et al., 2020), as well as suppressed glial reactivity (Meyer et al., 2018) in the ALS-mimic mouse model. Our RNAi experiment in the fly model also demonstrated that inhibition of *ERR* (fly ortholog of *NR3C1*) strongly abolished eye degeneration (Score = −3, Figure 2C and Table 3), suggesting *NR3C1* as an actionable target for ALS.

#### MAP3K5

Upregulation of *MAP3K5* was observed in 80% of fALS comparisons and 71% of sALS comparisons in CNS tissue. Current drugs targeting MAP3K5 were not tested in neurological disease. RNAi against *Ask1* (fly ortholog of *MAP3K5*) moderately rescued eye degeneration (Score = −2, Table 3) in the c9ALS fly model, indicating *MAP3K5* was a potential therapeutic target for ALS. Notably, our findings aligned with previous study reporting that MAP3K5 inhibitors prolonged the survival of SOD1^mut^ mice (Fujisawa et al., 2016), and were supported by accumulating evidence indicating that activation of MAP3K5 may contribute to neurodegeneration (Nishitoh et al., 2002; Kadowaki et al., 2005; Hu et al., 2011; Lee et al., 2012). Elevated levels of MAP3K5 were also reported in lymphocytes from ALS patients (Mougeot et al., 2011) and motor neurons of SOD1 transgenic mice (Holasek et al., 2005). Additionally, other studies revealed the linkage between *SOD1* mutant and MAP3K5 activation in neuronal cell death (Nishitoh et al., 2008; Lee et al., 2014), further supporting our observations.

#### ERN1

*ERN1*, encoding IRE1, was upregulated in diMN transcriptomic fALS samples (LFC = 0.2058, *p* = 0.003). The suppression of *Ire1* in flies impeded eye degeneration (Score = −2, Table 3), supporting *ERN1* as another potential target for ALS. IRE1 is one of the primary sensors for unfolded protein response (UPR), which serves as a critical stress response that copes with endoplasmic reticulum (ER) stress and maintains cell viability. IRE1 signaling was considered to be pathogenic in ALS (Montibeller and de Belleroche, 2018), Alzheimer’s disease (AD) (Duran-Aniotz et al., 2017) and PD (Yan et al., 2019). In SOD1^G93A^ mice, IRE1 protein level was elevated with disease progression (Chen et al., 2015). Moreover, administration of PPAR agonist exerted its protective effect on neurodegeneration through suppression of IRE1-mediated ER stress response (Tong et al., 2016), further backing our results.

#### *KCNB2* and *KCNS3*

*KCNB2* and *KCNS3*, two members of the voltage-gated potassium channel (Kv) family, were upregulated in CNS sALS (83%) comparisons and diMN transcriptomic sALS samples (LFC = 0.3338, *p* = 0.0282), respectively. In agreement with our results, the inhibition of *Shab* (fly ortholog of *KCNB2* and *KCNS3*) substantially hampered eye degeneration in the fly model (Score = −3.5, Figure 2C and Table 3). Apart from playing a key role in modulating neuronal excitability, Kv channels are also involved in cell cycle progression, proliferation, and apoptosis (Bachmann et al., 2020). It was suggested that *KCNB2* was one of the genes with the most significant copy number gains in ALS (Morello et al., 2018). *KCNS3* was reported to be upregulated in early pathological stages of AD (Saura et al., 2015), and as a risk gene in PD (Perrone et al., 2021). Administration of 4-Aminopyridine, a non-selective blocker of Kv channels, restored ion channel dynamics, rescued neuronal activity, and relieved ER stress in ALS MNs (Naujock et al., 2016), reinforcing our hypothesis.

#### P2RY14

*P2RY14* was downregulated in 86% (14% upregulated) of CNS sALS comparisons. It encodes a purinergic receptor responding to UDP-glucose and other UDP-sugars coupled to G-proteins, and provides a novel candidate for drug development. The role of P2RY14 in neurodegeneration remains unclear, as both neuroprotective and neurotoxic roles were reported. While it may execute its neuroprotective function by inhibiting the expression of MMP9 in AD (Erb et al., 2015), increased *P2RY14* expression was observed in rat primary LPS-mediated microglial activation (Bianco et al., 2005), correlating with neuroinflammation. Our results showed that suppressions of the two fly orthologs of *P2RY14* (*AstC-R1* and *AstC-R2*) by RNAis reduced eye degeneration in the c9ALS *Drosophila* model (Score = −3 for *AstC-R1*, Score = −2 for *AstC-R2*, Figure 2C and Table 3), suggesting that further investigation of P2RY14 function in neurodegeneration is warranted to confirm its relevance as potential target in ALS.

#### PPP3CB

*PPP3CB*, encoding the β-isoform of the catalytic subunit of Calcineurin (Cn), was selected as a potential target for ALS. Reduction of PPP3CB protein level was detected in diMN fALS samples (LFC = −0.3048, *p* = 0.0115). Cn stability depended on its interaction with SOD1 (Neurath et al., 1992). Weakening of SOD1^G93A^-Cn interaction in SOD1^G93A^ mice decreased Cn stability, leading to the defect in TDP-43 dephosphorylation and TDP-43 aggregation (Kim et al., 2019). Activation of PPP3CB/Cn stimulated the activity of transcription factor EB, and eventually promoted autophagy to ameliorate neurodegeneration (Rusmini et al., 2019). RNAis against *Pp2B-14D* and *CanA-14F* (two fly orthologs of *PPP3CB*) exerted suppressive effects (Score = −3 for *Pp2B-14D*, Score = −2 for *CanA-14F*; Figure 2C and Table 3) on fly eye degeneration, which indicated that perturbation of *PPP3CB* had functional correlations with neurodegeneration in ALS.

### Validation of AI-Based Target Discovery in a c9ALS *Drosophila* Model

To validate whether the targets we have identified were relevant to the disease, we used the c9ALS *Drosophila* model of ALS (Zhang et al., 2015). This model is based on the over-expression of a GGGGCC (G_4_C_2_) hexanucleotide repeat expansion (HRE) in *C9orf72*, the most common driver of ALS (DeJesus-Hernandez et al., 2011; Renton et al., 2011). Previously, we showed that expression of 30 repeats of G_4_C_2_ [(G_4_C_2_)_30_] using the UAS/GAL4 system, under the control of GMR-GAL4 (Brand and Perrimon, 1993), induced progressive neurodegeneration in *Drosophila* eyes, as indicated by defects in the external eye morphology (Zhang et al., 2015). Using this model, we have previously performed several RNAi screens (Zhang et al., 2015; Neuro et al., 2021), which identified many genes whose loss modifies (G_4_C_2_)_30_-mediated eye degeneration. To validate our findings, we compared the candidates identified by the PandaOmics analysis and our fly screening results. The severity of eye degeneration was assessed using the scoring scale shown in Figure 2A. As summarized in Table 3, the 28 candidate human genes correlated to 34 orthologs in the fly. No fly models were available for *SLC25A10* and *METTL21A*. Suppression of 18 of these 26 targets using RNAi strongly or moderately rescued eye degeneration (Table 3 and Figure 2B), suggesting that these genes may contribute to (G_4_C_2_)_30_-mediated neurotoxicity. On the other hand, depletion of *S6k* (fly ortholog of *RPS6KB1*) moderately enhanced eye degeneration. Representative images of fly eyes whose degenerations were strongly rescued by RNAi are shown in Figure 2C.

### Clustering of Dysregulated Pathways

The Reactome database provides the hierarchical organization of signaling pathways grouped into broader domains of biological functions (Sidiropoulos et al., 2017). Therefore, all the pathways in our analysis here were classified into 27 biological processes, each of which corresponded to one top-level pathway according to the Reactome hierarchy. In CNS groups, dysregulated pathways in ALS patients were overrepresented in the immune system process (fALS, adjusted *p* = 3.26E-7), signal transduction process (sALS, adjusted *p* = 9.20E-5), and hemostasis (fALS, adjusted *p* = 0.0054). The diMN transcriptomic groups were enriched for dysregulated pathways belonging to the protein metabolism process (sALS, adjusted *p* = 0.0093). The dysregulated pathways in the diMN proteomic groups were overrepresented in the processes of disease (sALS, adjusted *p* = 3.38E-8), DNA repair (fALS, adjusted *p* = 0.0233), and developmental biology (fALS, adjusted *p* = 0.0255). The details of dysregulated pathways in different biological processes are shown in Supplementary Table 2.

**FIGURE 3:**
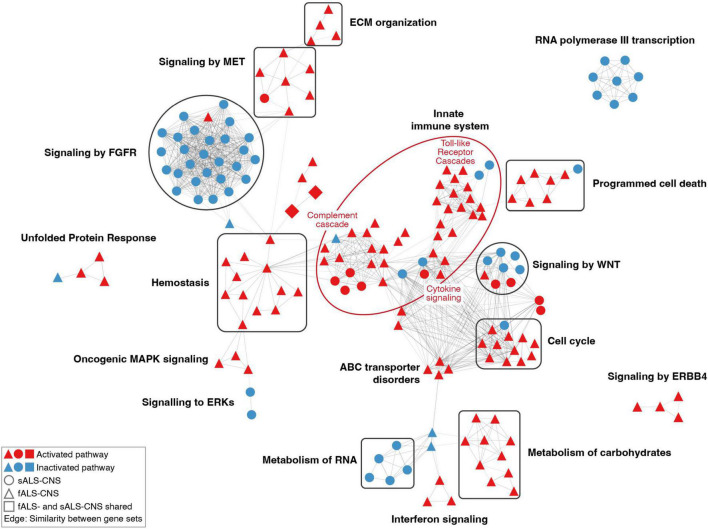
Network of dysregulated pathways in CNS comparisons. Each node represented a dysregulated pathway consisting of a set of genes. Nodes with similar gene contents (similarity coefficient > 0.35) were connected by edges, and the thickness of node-linking edges was proportional to the similarity between a pair of gene sets. Clusters of pathways were annotated based on the hierarchical level of pathways retrieved from the Reactome database. The constituent pathways of a cluster were colored in red or blue for activation or inactivation. Only clusters containing more than three pathways were shown.

Furthermore, pathways with similar gene contents were connected to form clusters. As shown in Figure 3, the most prominent cluster of the dysregulated pathways in CNS ALS groups (relative to healthy cohort) was associated with the activated innate immune system, which consisted of activated pathways of the Toll-like receptor cascades, cytokine signaling, and regulation of complement cascade. Several clusters of pathways known to be associated with ALS pathogenesis were also identified, including activated pathways of programmed cell death, unfolded protein response, and ERBB4 signaling. Other activated clusters included pathways of the extracellular matrix organization, MET signaling, hemostasis, oncogenic MAPK signaling, ABC transporter disorders, interferon signaling, carbohydrate metabolism, and cell cycle pathways associated with G1-S DNA damage checkpoint. Whereas pathways related to FGFR signaling, RNA metabolism, and RNA polymerase III transcription were inhibited. In addition, pathways of RNA polymerase I and II transcription, mitochondrial protein import, and NCAM signaling for neurite out-growth were also suppressed (Supplementary Table 3). Notably, there were only a few dysregulated pathways overlapping between fALS and sALS groups (i.e., upregulated pathways of erythropoietin activated PI3-kinase annotated as squares in Figure 3), and most clusters were specific to a sole ALS subtype. For example, clusters of innate immune system, hemostasis, carbohydrates metabolism, and ERBB4 signaling mainly contained activated pathways identified in fALS but not the sALS; while the clusters of FGFR signaling, RNA metabolism, and RNA polymerase III transcription mainly contained inhibited pathways identified in the sALS only.

**FIGURE 4:**
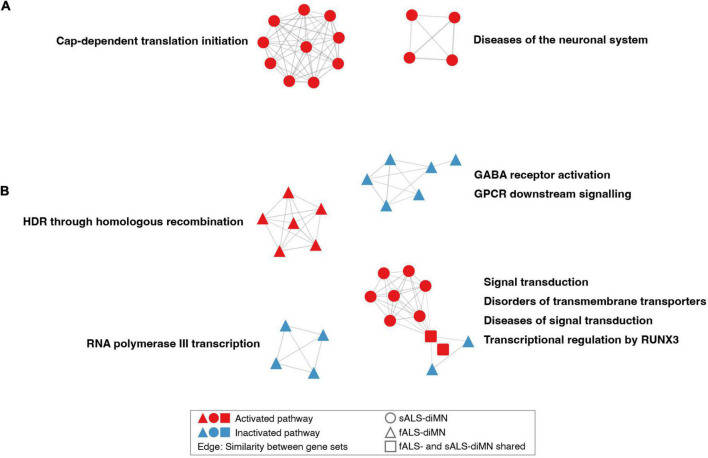
Network of dysregulated pathways in diMN comparisons. Dysregulated pathways based on diMN **(A)** transcriptomic and **(B)** proteomic comparisons. Notations refer to Figure 3. Only clusters containing more than three pathways were shown.

The dysregulated pathways in the diMN ALS groups belonged to different biological processes when compared to the CNS groups. For the diMN transcriptomic comparisons, pathways of the cap-dependent translation, and diseases of the neuronal system were found to be activated in ALS case groups (Figure 4A). For the diMN proteomic comparisons, the RNA polymerase III transcription, GABA receptor, and GPCR signaling pathways were found to be inhibited (Figure 4B). On the other hand, pathways of DNA homologous recombination repair were activated. The pathways related to signal transduction and its related diseases, transmembrane transporter disorders, and transcriptional regulation by RUNX3 formed the largest cluster due to their shared genes of the ubiquitin-proteasome system, such as the ubiquitin genes (*UBC*, *UBB* and *UBA52*), the proteasome genes (*SEM1*, *RPS27A*, and PSM subunits), and the ER-associated degradation genes (*VCP*, *SEL1L*, *OS9*, *ERLEC1*, and *DERL2*).

## Discussion

After decades of research, the genetic and environmental factors contributing to the etiology of ALS remain inadequately defined. Integrative multi-omics approaches have been employed to dissect the disease pathophysiology (Ruffini et al., 2020; Volonte et al., 2020; Straub et al., 2021). PandaOmics is a fully integrated AI-based platform with a wide range of omics and text data sources (Vera et al., 2022). Compared to other existing tools for target discovery, PandaOmics has several unique advantages with respect to user experience, algorithms, the comprehensive database, and the time machine validation approach (Zhavoronkov et al., 2019). In an easy to use manner, this platform is able to define druggable targets using multiple advanced bioinformatics and AI models, accelerating the drug discovery process (Insilico Medicine, 2022; Pun et al., 2022). Therefore, PandaOmics represents a unique and user-friendly AI-driven target discovery platform for therapeutic target exploration based on multi-omics data analysis, that requires no prior knowledge of computational biology.

With the advance of medical care and improved lifestyles, human life expectancy has been significantly lengthened, which in turn poses significant health-associated challenges, due to the shift in demographic structure toward the aged. Despite multiple risk factors being proposed to contribute to ALS, aging remains one of the most prevalent risk factors and driving forces for developing the disease (Pandya and Patani, 2020). Identifying dual-purpose targets implicated in both aging and ALS is an intriguing geroscience approach for extending healthspan and delaying age-associated health issues (Melov, 2016). Among the 28 shortlisted therapeutic targets identified in this study, 8 (28.6%, marked with asterisks in Table 2) were suggested to be aging-associated based on the evidence from clinical trials^2^, publications, geroprotectors^3^ and GenAge database, indicating the association between aging and ALS.

**FIGURE 5:**
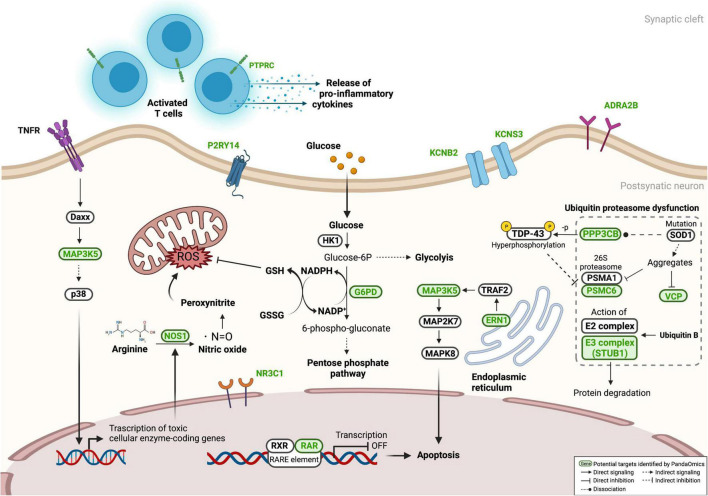
Proposed targets in ALS-associated pathways. Several proposed targets, labeled in green, were presented in pathways related to ALS pathogenesis, including neuronal cell death, oxidative stress, neuroinflammation, and proteostasis dysfunction. Information of associated networks was retrieved from KEGG ALS pathway (map05014), Protein processing in endoplasmic reticulum (map04141), T cell receptor signaling pathway (map04660), Ubiquitin mediated proteolysis (map04120), and Pentose phosphate pathway (map00030).

As summarized in Figure 5, therapeutic targets identified in our work were mainly associated with the two fundamental cellular processes in the pathogenesis of ALS – proteostasis dysfunction and neuronal death. Twenty-six targets were validated in the c9ALS *Drosophila* model, of which eighteen demonstrated that their depletion has rescued neurodegeneration, while the loss of *RPS6KB1* resulted in an opposite effect. This validation confirmed the power of PandaOmics in identifying therapeutic targets with potential roles in ALS neurodegeneration. Although some of the proposed targets were not directly associated with neurodegeneration, all of them have been reported to participate in pathways that may contribute to ALS development. Some well-known ALS-associated genes, such as *TARDBP*, *C9orf72* and *FUS*, were not included in our target list, as they did not belong to any druggable classes and thus were filtered out. *SOD1*, ranked among top 10 in the high-confidence genes of diMN proteomic meta-analyses, was also not proposed in the present study, as its role in ALS pathology is well-established. Overall, we demonstrated our AI-enabled target discovery approach in accelerating the novel ALS target discovery process for new therapeutic regimen development.

We showed that several enriched pathway clusters were closely linked with ALS-driven mechanisms. For example, RNA metabolism was commonly dysregulated in our analysis regardless of tissue type. Altered RNA metabolism was indicated as a key concern in ALS (Zaepfel and Rothstein, 2021). Several research groups have evaluated the relevance of ALS genes to RNA metabolism, revealing that mutations in TDP-43 (Tollervey et al., 2011; Barmada et al., 2015; Russo et al., 2017; Donde et al., 2019; Coyne et al., 2021), FUS (Barmada et al., 2015) and C9orf72 (Donnelly et al., 2013; Lee et al., 2013) induce pathogenic RNA metabolic changes in ALS. It is noteworthy that there were many discrepancies between CNS and diMN pathway networks. Pathways controlling innate immune response and programmed cell death were found to be upregulated in our CNS comparisons. The hemostasis and erythropoietin signaling pathways were also upregulated, suggesting an activated neuro-immune hemostasis network in response to the CNS tissue damages (De Luca et al., 2018). These results are in agreement with the properties of post-mortem CNS tissues, consisting of miscellaneous cell types, such as neurons, glial cells, as well as CNS-resident and infiltrated immune cells upon neuronal injury. Such that the observed inflammatory disturbance in the ALS CNS tissues reflected the late-stage phenotype in ALS or a general phenomenon in the dying brain. Conversely, diMN ALS samples were solely derived from motor neurons, without the influence of non-neuron cells and the aging process. Such comparisons clearly reflected the disease pathology in motor neurons. We also showed that GABAergic signaling pathways were downregulated. This may lead to an increase in glutamate toxicity (Foerster et al., 2012; Romano et al., 2021). Excitotoxicity, a pathophysiological condition caused by excessive glutamate stimulation, is suspected as a mediator driving ALS development (Van Den Bosch et al., 2006; Armada-Moreira et al., 2020). GABAergic signaling may function to counteract excessive neuronal excitability, inducing a calming effect. Furthermore, pathways in homologous recombination, a DNA damage response, and cap-dependent translation initialization, a component in the RNA metabolism, were dysregulated in diMN comparisons but not CNS. These processes are likely to contribute to ALS initiation (Sun et al., 2020), indicating that the diMN comparisons revealed early-stage signatures of disease development. A recent study investigating Alzheimer’s progression in the human brain highlighted the importance of integrating human data with data generated using cell lines and animal models, in order to better understand various stages of disease evolution (Penney et al., 2020). As such, our usage of post-mortem CNS tissue as well as diMN samples provides a more detailed view of ALS pathogenesis.

It is not surprising that fewer pathways were uniformly altered in sALS relative to fALS comparisons given the complex genetic bases and the large variability among sALS individuals (Figure 3). However, there were some pathway clusters that are specific to sALS, such as the FGFR signaling axis. Fibroblast growth factors and their receptors play essential roles in the development, maintenance and repair of the nervous system (Reuss and von Bohlen und Halbach, 2003; Maddaluno et al., 2017; Klimaschewski and Claus, 2021). The inhibition of FGFR signaling indicates the reduction of neurogenetic effects underlying ALS etiology, which was confirmed in the CNS sALS groups (Figure 3). However, it was not observed in the CNS fALS groups, which might stem from the lack of association between FGF signaling and *C9orf72* mutations that represent the dominant genotype in the fALS comparisons (Table 1).

The current study has a limited number of fALS samples in both post-mortem and diMN comparisons due to the rarity of fALS incidence. Another limitation of our analysis is the under-representation of racial groups other than the Caucasians. Future studies should include samples from more ethnically heterogeneous populations. ALS is a progressive disorder, driven by numerous interconnected mechanisms. While future studies are warranted to assess the pathogenic mechanisms underlying every stage of disease development, data generated in the current analysis is likely representing two stages in ALS development - the late-stage in post-mortem CNS tissue, and the early-stage reflected in diMN samples, harvested on Day 32. This time frame is generally adopted as the maturation time point of diMNs (Du et al., 2015; Guo et al., 2017; Solomon et al., 2021); therefore, this study model might not well represent the aging effect on ALS disease progression. Including additional diMN time points throughout the developmental stages of the disease might enhance the comprehensiveness of the analysis and provide a more detailed insight on ALS progression.

In conclusion, this study demonstrates the application of PandaOmics target discovery system to identify and prioritize high-confidence and novel targets for ALS with our latest AI models based on comprehensive omics data analysis. Several well-characterized mechanisms in ALS pathology were found to be dysregulated, including the immune system, RNA metabolism, excitotoxicity, as well as programmed cell death. Seventeen high-confidence and eleven novel therapeutic targets were identified from CNS and diMN samples. CNS data mainly reflects the late-stage signatures of ALS, while results from diMN comparisons are more likely to be attributed to the early-stage signatures. Combining the usage of diMN and post-mortem CNS samples could provide a comprehensive understanding of ALS disease progression. The employment of the *Drosophila* model exemplified a fast screen of AI-identified targets. Among the 26 proposed targets screened in the c9ALS *Drosophila* model, we were able to verify 8 unreported genes (*KCNB2*, *KCNS3*, *ADRA2B*, *NR3C1*, *P2RY14*, *PPP3CB*, *PTPRC*, and *RARA*) whose suppression strongly rescued eye neurodegeneration. Future studies are warranted to further define their pathogenic role and potential as therapeutic targets for c9- and other types of ALS using diMNs or mammalian models. To accelerate novel target discovery and drug investigation for ALS, targets identified in this study will be disclosed on ALS.AI. Altogether, the present study offers new insights on how AI speeds up the target discovery process from years to months.

## Data Availability Statement

The original contributions presented in this study are included in the article/Supplementary Material, further inquiries can be directed to the corresponding author/s.

## Ethics Statement

Ethical review and approval were not required as only public available human data and no animal experiments were involved in the study. ALS data were obtained with written consent from every ALS and control patient.

## Author Contributions

FP performed the data analysis, result interpretation, project administration, and writing the original draft. BLi and XL conducted the data analysis, visualization, result interpretation, and writing the original draft. HL performed the result interpretation and validation. GL conducted the result interpretation and website design. QM performed the fly experiments and result interpretation, and wrote the manuscript. JG performed the fly experiments and result interpretation. AS conducted the data curation and processing. IO performed the data curation, methodology and software. JW, FR, and XG performed result interpretation. AA was responsible for methodology and resources. EB and EI performed result interpretation and reviewed the manuscript. KZ provided conceptualization, funding acquisition, supervision for the fly experiments, and wrote the manuscript. BLu performed result interpretation and reviewed the manuscript. JR and MC conducted the data acquisition, result interpretation and reviewed the manuscript. AZ provided the conceptualization, resources, and supervision. All authors have read and agreed to the published version of the manuscript.

## Conflict of Interest

FP, BLi, XL, HL, GL, AS, IO, JW, FR, AA, and AZ were employed by Insilico Medicine Hong Kong Ltd. XG was employed by 4B Technologies Limited. The remaining authors declare that the research was conducted in the absence of any commercial or financial relationships that could be construed as a potential conflict of interest. The handling editor AS declared a shared parent affiliation with one the authors, MC at the time of the review.

## Publisher’s Note

All claims expressed in this article are solely those of the authors and do not necessarily represent those of their affiliated organizations, or those of the publisher, the editors and the reviewers. Any product that may be evaluated in this article, or claim that may be made by its manufacturer, is not guaranteed or endorsed by the publisher.

## Footnotes

1
http://als.ai/

2
https://clinicaltrials.gov/

3
http://geroprotectors.org

## Abbreviations

AD: Alzheimer’s disease
AI: Artificial intelligence
ALS: Amyotrophic lateral sclerosis
fALS: Familial ALS
sALS: Sporadic ALS
c9ALS: *C9orf72*-mediated ALS
CNS: Central nervous system
Cn: Calcineurin
diMN: Direct iPSC-derived motor neuron
ER: Endoplasmic reticulum
HRE: Hexanucleotide repeat expansion
iPSC: Induced pluripotent stem cell
KOL: Key opinion leader
Kv: voltage-gated potassium channel
LFC: log2-transformed fold change
MN: Motor neuron
PD: Parkinson’s disease
RNA-seq: RNA sequencing
UPR: Unfolded protein response.
