# Supplementary material for: Identification of Therapeutic Targets for Amyotrophic Lateral Sclerosis Using PandaOmics – An AI-Enabled Biological Target Discovery Platform
Source: Front Aging Neurosci. 2022 Jun 28;14:914017. doi: 10.3389/fnagi.2022.914017 (PMC9273868; doi:10.3389/fnagi.2022.914017)
Supplement: Supplementary file 1 [file Data_Sheet_1.pdf]

Supplementary Material

1 Captions for Supplementary Tables

Supplementary Table 1. Description for scores and filters in PandaOmics.

Supplementary Table 2. Enrichment of dysregulated pathways in different biological processes.

Supplementary Table 3. Dysregulated pathways in CNS, diMN transcriptomic and diMN proteomic groups.

2 Supplementary Figures

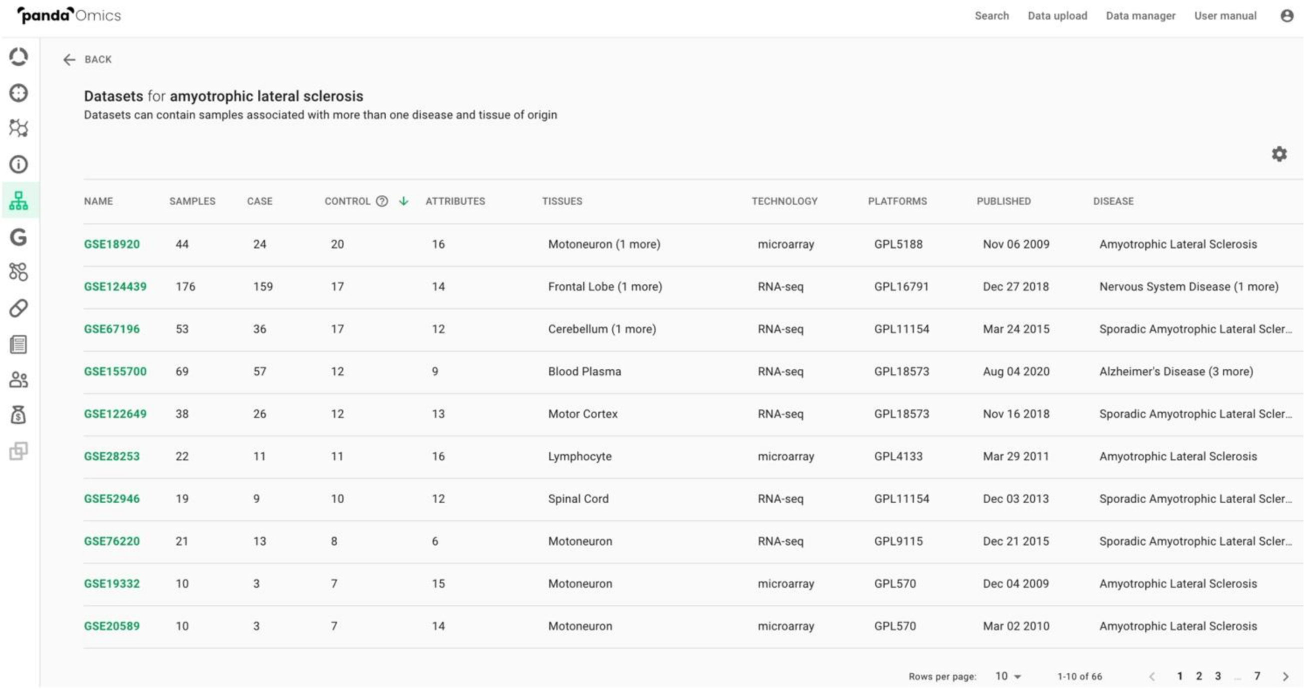

NAME	SAMPLES	CASE	CONTROL	ATTRIBUTES	TISSUES	TECHNOLOGY	PLATFORMS	PUBLISHED	DISEASE
GSE18920	44	24	20	16	Motoneuron (1 more)	microarray	GPL5188	Nov 06 2009	Amyotrophic Lateral Sclerosis
GSE124439	176	159	17	14	Frontal Lobe (1 more)	RNA-seq	GPL16791	Dec 27 2018	Nervous System Disease (1 more)
GSE67196	53	36	17	12	Cerebellum (1 more)	RNA-seq	GPL11154	Mar 24 2015	Sporadic Amyotrophic Lateral Scler...
GSE155700	69	57	12	9	Blood Plasma	RNA-seq	GPL18573	Aug 04 2020	Alzheimer's Disease (3 more)
GSE122649	38	26	12	13	Motor Cortex	RNA-seq	GPL18573	Nov 16 2018	Sporadic Amyotrophic Lateral Scler...
GSE28253	22	11	11	16	Lymphocyte	microarray	GPL4133	Mar 29 2011	Amyotrophic Lateral Sclerosis
GSE52946	19	9	10	12	Spinal Cord	RNA-seq	GPL11154	Dec 03 2013	Sporadic Amyotrophic Lateral Scler...
GSE76220	21	13	8	6	Motoneuron	RNA-seq	GPL9115	Dec 21 2015	Sporadic Amyotrophic Lateral Scler...
GSE19332	10	3	7	15	Motoneuron	microarray	GPL570	Dec 04 2009	Amyotrophic Lateral Sclerosis
GSE20589	10	3	7	14	Motoneuron	microarray	GPL570	Mar 02 2010	Amyotrophic Lateral Sclerosis

Rows per page: 10 · 1-10 of 66 · 1 2 3 ... 7

Supplementary Figure 1. ALS-related datasets in PandaOmics. Sixty-six transcriptomic datasets associated with ALS were extracted from public repositories (e.g. Gene Expression Omnibus and ArrayExpress) and processed for standardization before uploading onto PandaOmics. Number of total samples (as well as cases and controls), tissue type, technology and platform used, and published date were listed in the Dataset page for ALS.

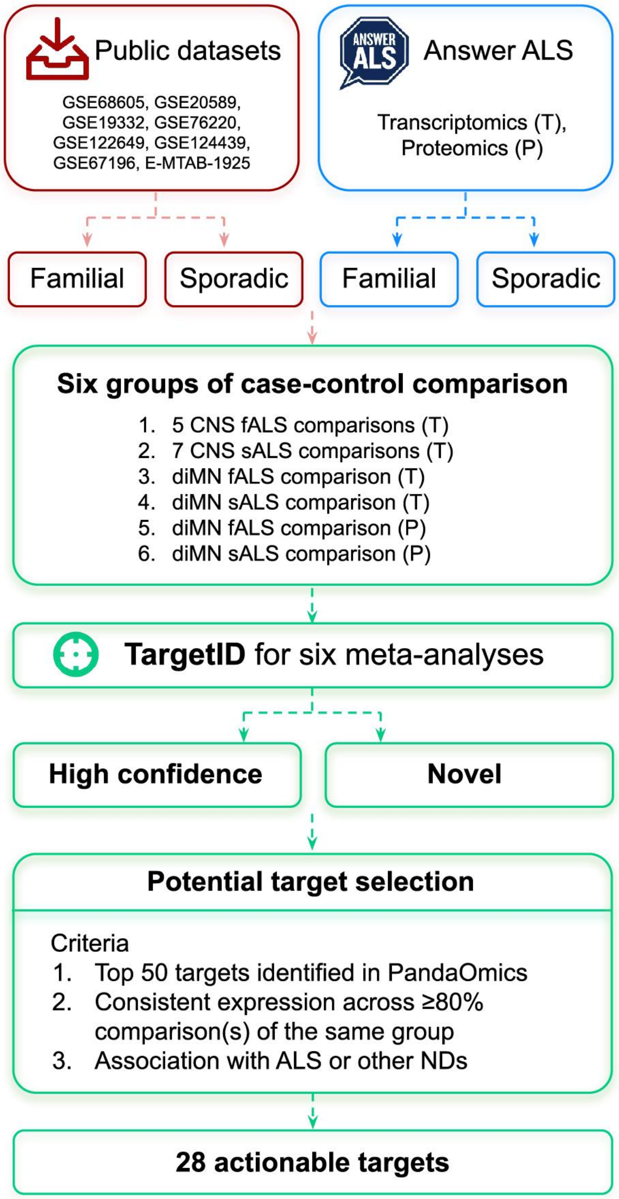

Supplementary Figure 2. Schematic workflow for target identification. Eight transcriptomic datasets using post-mortem CNS samples retrieved from public repositories, along with the transcriptomic and proteomic data using diMN samples from Answer ALS were analyzed in the present study. For each dataset, the ALS patients were divided into the familial and sporadic subtypes based on the family history of ALS occurrence or the presence of ALS subtype-linked gene mutations. Comparisons were made between case and control samples independently for different tissues, ALS subtypes and data types. To identify potential targets, all the case-control comparisons belonging to the same comparison group were pooled into a single meta-analysis, yielding a total of six meta-analyses. For each meta-analysis, PandaOmics prioritized the targets under two novelty setting (viz. high-confidence and novel settings specified in Methods). Potential therapeutic targets were further selected with three criteria, yielding a total of 28 actionable targets. T: Transcriptomics; P: Proteomics; ND: Neurodegenerative disease.

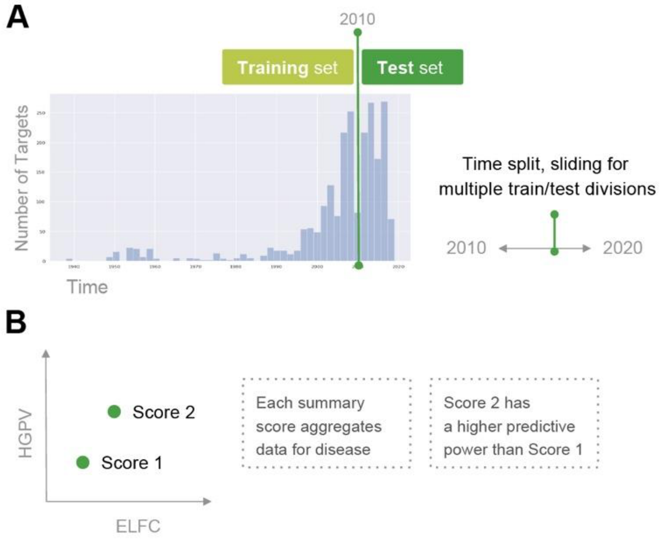

Supplementary Figure 3. Schematic representation of scoring-approach validation. To validate the models of novel target identification, the "time machine" approach was applied. **(A)** Taking year 2010 as an example time split, clinical data before the year was used as the training set and that after was the testing set. **(B)** Each scoring approach was validated by ELFC (log fold change of enrichment) and HGPV (significance of the enrichment). Its predictive power increases with the rise of ELFC and HGPV values.

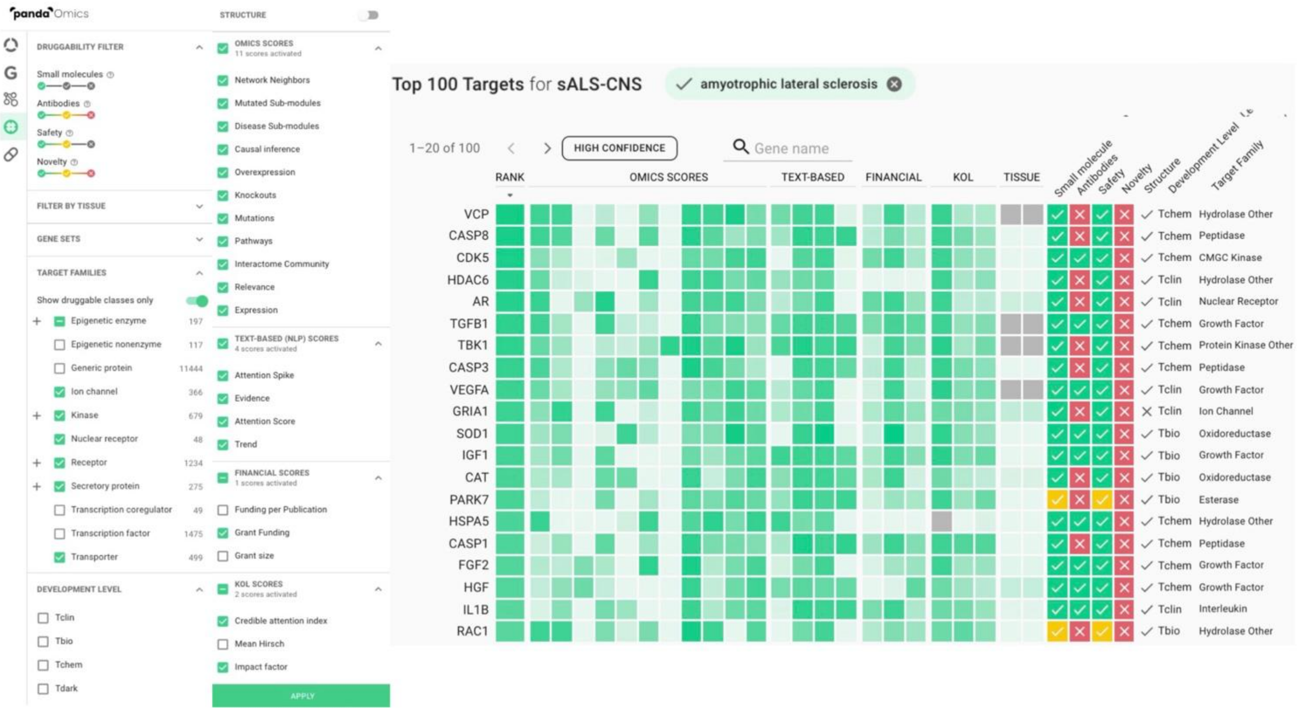

Supplementary Figure 4. Filter setting for high-confidence targets identification. With the use of over 20 AI and bioinformatics models, PandaOmics ranks related genes and identifies potential targets based on twenty-one scores from Omics, Text-based, Financial and KOL categories, as well as the Druggability filters. To identify high-confidence druggable targets, eighteen scores and Druggability Classes were applied. Druggability filters were also customized to select targets with associated small molecules and medium safety levels.

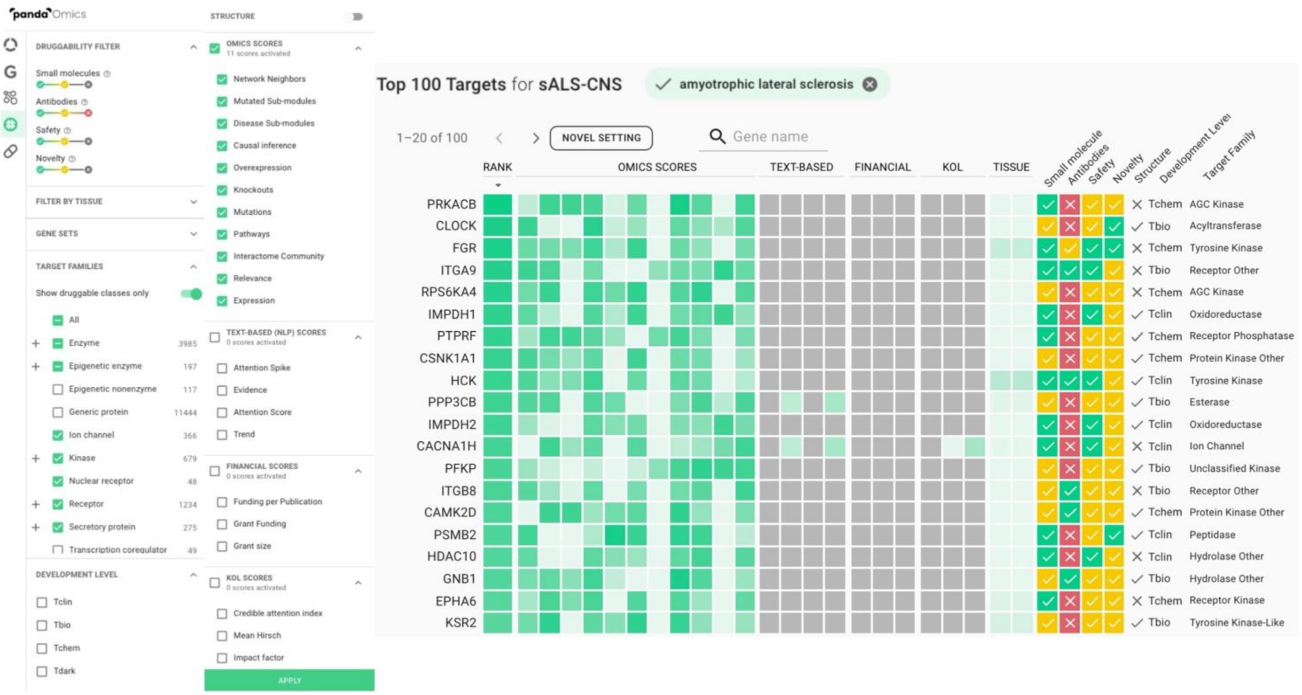

Supplementary Figure 5. Filter setting for novel targets identification. Novel targets are defined as druggable targets without prior knowledge in ALS. To achieve this criterion, the Druggability filter was restricted to a higher novelty level, and only Omics scores were selected for the recalculation of the metascores.

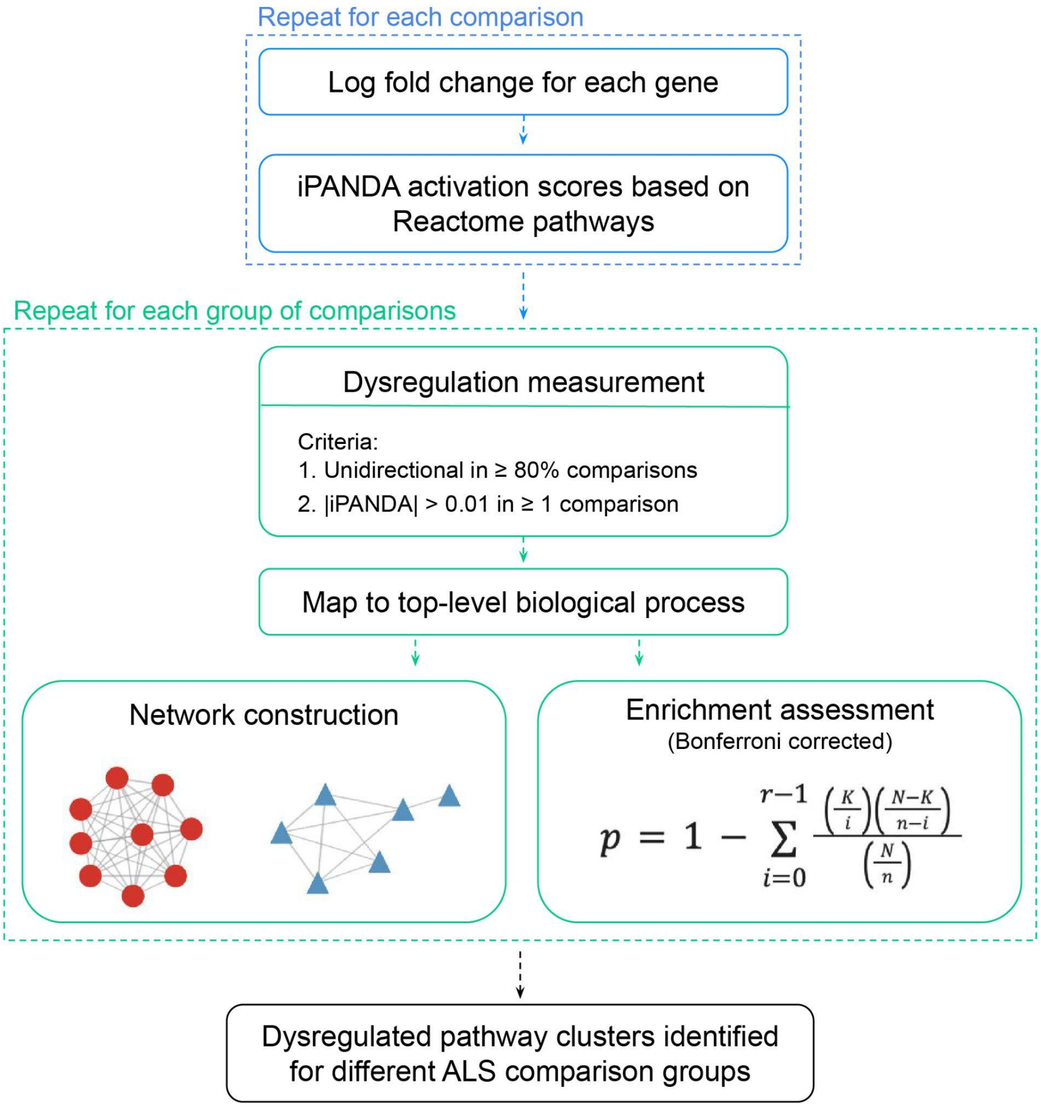

Supplementary Figure 6. Schematic flow for pathway analysis. For each ALS comparison, the log-transformed fold changes between the gene expression levels in the case and control samples were used as the input data for the iPANDA algorithm, yielding positive iPANDA values for activated pathways and negative values for inactivated pathways. Then, the dysregulation of pathways was measured in each of the six groups of comparisons respectively. Taken the CNS fALS group (number of comparisons = 5) as an example, a pathway was considered as dysregulated when 1) its alteration was unidirectional in greater than or equal to 80% of all the comparisons (viz. ≥ 4 out of the 5 comparisons), and 2) the absolute iPANDA value reached the threshold of 0.01 in at least one comparison. Next, the dysregulated pathways were mapped to higher-level biological processes based on the hierarchical level of pathways retrieved from the Reactome database. Finally, dysregulated pathways with shared genes were connected to form the network of dysregulated pathways. In addition, the significant level of the enrichment of dysregulated pathways in different top-level biological processes were evaluated using hypergeometric tests.
